# Regulatory T cells in hypoxic environments

**DOI:** 10.3389/fimmu.2026.1755928

**Published:** 2026-02-24

**Authors:** Lourdes González-Rivera, Rodrigo Luna-Gutiérrez, Stephanie Cárdenas, Consuelo Merino-González, Alex Handy, Inés Pepper, Mercedes Natalia López

**Affiliations:** 1 Facultad de Medicina, Instituto de Ciencias Biomédicas (ICBM), Universidad de Chile, Núcleo Interdisciplinario de Farmacología e Inmunología, Santiago, Chile; 2 Departamento de Inmunología, Hospital Clínico Universidad de Chile José Joaquín Aguirre, Santiago, Chile; 3 Instituto de Ciencias Biomédicas (ICBM), Facultad de Medicina, Universidad de Chile, Núcleo Interdisciplinario de Fisiología, Biofísica y Fisiopatología, Santiago, Chile; 4 Faculty of Medicine, Universidad de Chile, Santiago, Chile

**Keywords:** regulatory T (Treg) cell, hypoxia-Inducible factor 1, alpha Subunit, hypoxia, metabolic reprogramming, obstructive sleep apnea, chronic obstructive pulmonary disease, COVID-19, inflammatory mediators

## Abstract

Oxygen availability is considered as an important determinant of immune regulation, yet its impact on regulatory T cells remains incompletely understood. In this review, we synthesize current evidence on how chronic and intermittent hypoxia influence the differentiation, stability and function of regulatory T cells across diverse physiological and pathological settings. We describe the main cellular pathways engaged during hypoxic adaptation, with emphasis on the role of hypoxia-inducible factors in shaping regulatory T cell metabolism and lineage integrity. We then evaluate findings from clinical contexts characterized by sustained or cyclical oxygen deprivation, including chronic lung disease, sleep-disordered breathing and severe viral infection. Across these conditions, hypoxia is associated with alterations in regulatory T cell phenotype and its suppressive function, although patterns vary according to microenvironment and disease stage. A clearer understanding of how distinct hypoxic patterns modulate regulatory T cell biology will be essential for identifying therapeutic strategies aimed at restoring immune balance in hypoxia-associated disease.

## Introduction

Regulatory T cells (Tregs) maintain immune homeostasis and self-tolerance by restraining autoreactive and inflammatory responses (1), a concept that emerged from early tolerance studies and the historical “suppressor T cell” paradigm (2). A landmark study by Sakaguchi et al. identified CD4^+^ cells with high, sustained CD25 (IL-2Rα) expression as essential for self-tolerance, establishing CD25 as an early Treg marker (3). The subsequent identification of FOXP3 as a lineage-defining transcription factor was pivotal; in parallel genetic and functional studies, Brunkow et al. and Wildin et al. demonstrated that FOXP3 is indispensable for immune regulation in mice and humans (4, 5), providing a mechanistic explanation for immune dysregulation, polyendocrinopathy, enteropathy, X-linked (IPEX) syndrome due to pathogenic FOXP3 variants (5, 6). Subsequent studies showed that FOXP3 is preferentially expressed in CD4^+^CD25^+^ cells and is sufficient to confer suppressive function, establishing it as the master regulator of the Treg lineage (7, 8).

Tregs are classified by developmental origin into thymus-derived Tregs (tTregs) and induced Tregs, the latter arising from naïve conventional CD4^+^ T cells upon T cell receptor activation under regulatory polarization conditions, most prominently TGF-β and IL-2 (9, 10). Induced Tregs arise either *in vivo* in peripheral tissues (pTregs) or *in vitro* under experimental polarization (iTregs) (9, 11). pTregs are generated in peripheral tissues and can acquire tissue-adapted features shaped by local signals (9, 12). At the epigenetic level, pTregs display partial demethylation of the Treg-specific demethylated region (TSDR), in contrast to the fully demethylated TSDR observed in tTregs, which confers greater stability of FOXP3 expression (13, 14). Consistent with their mode of generation, iTregs reflect the *in vitro* T cell receptor stimulus, whereas pTregs are enriched for environmental antigens and support tolerance to commensal microbiota (15, 16).

Beyond immune suppression, Tregs regulate immune homeostasis through multiple mechanisms, including cytokine secretion, modulation of antigen-presenting cells, metabolic interference, and cytolysis (17). In addition to controlling inflammation, Tregs actively promote tissue repair and regeneration across multiple organs (18). This combined capacity for immunoregulation and tissue support is now recognized as a fundamental aspect of Treg biology, enabling these cells to preserve systemic and local homeostasis across a range of physiological contexts (19). However, under sustained inflammatory stress, Treg plasticity may drive partial lineage instability, giving rise to hybrid populations that co-express FOXP3 and effector transcription factors such as RORγt and can acquire Th17-like features (20, 21).

Among the many factors shaping Treg function in peripheral tissues, oxygen availability has emerged as a key regulator, with growing evidence indicating that it can influence their differentiation, stability, and suppressive capacity (22, 23). Defining how Tregs respond to hypoxia is fundamental for interpreting their immunoregulatory function in hypoxia-driven diseases. This review highlights current evidence from clinically relevant hypoxic conditions, with emphasis on obstructive sleep apnea, chronic lung disease, and COVID-19, where hypoxemia and inflammation converge to shape Treg phenotype and function.

## Hypoxia

Hypoxia arises when local oxygen delivery fails to meet metabolic demand (24, 25). This review focuses on chronic sustained (CH) and intermittent hypoxia (IH), excluding acute patterns. CH, observed in chronic obstructive pulmonary disease (COPD), involves long-lasting oxygen reduction that reshapes immune metabolism, gene expression, and function (23, 26). IH, characteristic of obstructive sleep apnea (OSA), consists of recurrent deprivation-reoxygenation cycles promoting oxidative stress and inflammatory signaling via redox-sensitive pathways (27). Importantly, analogous oxygen landscapes also emerge beyond cardiopulmonary disease: solid tumors display spatially heterogeneous hypoxic niches together with temporally fluctuating hypoxia within the tumor microenvironment (TME), conditions that can promote immunosuppression and immune evasion (28). Although hypoxia has been widely examined across inflammatory settings, its Treg-specific effects on differentiation, metabolic programming and suppressive function remain incompletely defined, underscoring the need to resolve how CH and IH differentially shape Treg adaptation.

## HIF pathway and cellular adaptation to hypoxia

The cellular response to reduced oxygen availability is largely coordinated by hypoxia-inducible factors (HIFs), which link oxygen sensing to transcriptional programs that regulate metabolism and inflammation (26). Two major isoforms operate in this pathway, HIF-1α and HIF-2α. While HIF-1α is broadly expressed, HIF-2α displays a more restricted distribution across immune cells (29). Functionally, HIF-1α predominates in early/acute hypoxia, while also influencing T cell differentiation and the balance between effector and regulatory lineages (30, 31). By contrast, HIF-2α becomes more prominent during sustained hypoxia (32). Mechanistically, HIF acts as an alpha-beta heterodimer that binds hypoxia-response elements to induce adaptive gene expression (23). In normoxia, prolyl hydroxylase enzymes hydroxylate HIF-1α, enabling Hippel-Lindau (VHL) dependent ubiquitination and proteasomal degradation; when oxygen becomes limiting, hydroxylation is inhibited, allowing HIF stabilization, nuclear accumulation, and activation of programs linked to glycolysis, angiogenesis, and survival (25, 29, 33).

Beyond its canonical role as an oxygen sensor, HIF-1α has emerged as a central regulator of immunometabolism, a field that explores the interplay between metabolic pathways and immune function (22, 34). Under hypoxic stress, HIF-driven transcriptional programs redirect metabolic flux from mitochondrial oxidative phosphorylation (OXPHOS) toward glycolysis, enabling energy production in the absence of sufficient oxygen (33, 35). In immune cells, metabolic reprogramming is not merely a survival mechanism but a determinant of functional identity, as differentiation and effector specialization rely on distinct metabolic pathways (36).

## Tregs and HIF

Despite increasing recognition of the interplay between oxygen availability and immune regulation, the mechanisms through which hypoxia and HIF-1α shape Treg differentiation and stability remain incompletely understood. Tregs reside and function within a wide range of low-oxygen microenvironments, including inflamed tissues, tumors, and mucosal barriers where they undergo phenotypic and metabolic adaptation (22, 37). In Tregs, HIF-2α is essential for suppressive activity; its deficiency promotes lineage instability, conversion into IL-17-producing ex-Tregs, and impaired inflammatory control. HIF-2α limits HIF-1α activity, stabilizing FOXP3 and restraining Th17 polarization under chronic hypoxic conditions (38).

A complementary example of sustained hypoxia shaping Treg biology is hypoxia-associated pulmonary vascular remodeling, where chronic hypoxia converges with IL-6-dominated inflammation and metabolic stress (39–41). In pulmonary hypertension models, impaired Treg stability and function is linked to amplified perivascular inflammation and remodeling, consistent with hypoxia-driven erosion of FOXP3-dependent regulation (38, 39, 42, 43). Mechanistically, this aligns with the pathways outlined above in which sustained hypoxic signaling and inflammatory cues reinforce glycolytic programming and epigenetic destabilization of the FOXP3 locus, thereby promoting polarization toward IL-17-producing phenotypes (38–40).

Treg bioenergetics are primarily supported by OXPHOS and fatty acid oxidation; however, in inflammatory niches, they can upregulate glycolysis for migration and survival (35). Experimental studies indicate that restricting glycolytic activity enhances Treg stability and expansion, highlighting a close link between metabolic state and suppressive function (35, 44). However, current evidence, particularly in humans, remains limited and heterogeneous, with evidence supporting both pro- and anti-regulatory effects of hypoxia on Treg differentiation and suppressive function (22, 36, 44). This variability likely reflects differences in hypoxic pattern, tissue context, inflammation, and metabolic demands.

This complexity is partly driven by the dual, context-dependent actions of HIF-1α, which functions as an intrinsic metabolic checkpoint that can oppose Treg lineage commitment and stability (22, 45). Under physiological or basal metabolic conditions, HIF-1α promotes a glycolytic program that is incompatible with the OXPHOS-dependent metabolic profile required for stable Treg differentiation (44). The inhibitory role of HIF-1α is executed primarily through a post-translational mechanism: HIF-1α can directly bind FOXP3 and target it for ubiquitination and proteasomal degradation, thereby reducing FOXP3 protein abundance without altering its transcription (46, 47).

This degradative mechanism, through which HIF-1α attenuates Treg development by binding FOXP3 and targeting it for proteasomal degradation, contrasts with its context-dependent, non-inhibitory role in certain physiological settings (45, 46). For instance, in the intestinal mucosa, HIF-1α binds the FOXP3 promoter to enhance transcription and support Treg function (25, 48). Beyond post-translational mechanisms, HIF-1α also influences Treg stability through epigenetic regulation of the FOXP3 locus (45). Sustained FOXP3 expression in committed Tregs depends on TSDR demethylation by the ten-eleven translocation (TET) enzymes. Hypoxia, in part via HIF-1α stabilization, limits TET activity, favoring TSDR hypermethylation and destabilizing FOXP3 transcription (20, 26). This is reinforced by IL-6/STAT3 signaling, which upregulates DNA methyltransferase 1 (DNMT1) to directly methylate the TSDR, antagonizing the program required for stable Treg identity (20). Together, these mechanisms create a feed-forward loop whereby hypoxia and inflammation synergize to erode Treg stability and promote Th17 polarization (45, 49).

Shi et al. first identified HIF-1α-driven glycolysis as a metabolic checkpoint that diverts CD4^+^ T cell differentiation toward Th17 cells while suppressing FOXP3^+^ Treg generation (35). Building on this, Dang et al. demonstrated that HIF-1α not only promotes RORγt activation but also binds directly to FOXP3, targeting it for ubiquitination and degradation, thereby restraining Treg differentiation under hypoxic or inflammatory conditions (45). However, the relevance of these pathways under hypoxia *in vivo* remains unclear. Single-cell analyses by Lantz et al. did not reveal differential HIF-1α expression in Tregs, yet suggested that hypoxia-induced stabilization of HIF-1α in FOXP3-low Tregs may promote their conversion toward Th17 cells via RORγt activation and FOXP3 destabilization (21).

The influence of HIF-1α is not uniformly suppressive. In mucosal hypoxia, HIF-1α can enhance FOXP3 transcription and support Treg function. Clambey et al. showed that physiological hypoxia promotes FOXP3 induction via direct HIF-1α binding to hypoxia-responsive elements in the FOXP3 promoter (48). Complementary work by Lee et al. demonstrated that the VHL-HIF regulatory axis dynamically controls Treg stability: constitutive HIF-1α activation destabilizes the lineage and reduces FOXP3 expression, whereas physiological HIF-1α activity supports Treg adaptation (46).

In this context, Feldhoff et al. demonstrated that pharmacological HIF-1α stabilization with dimethyloxalylglycine (DMOG) impairs *de novo* iTreg differentiation, reducing FOXP3^+^ frequency from ~40% to ~10% without redirecting cells toward Th17 or Th2 lineages. This supports the interpretation that HIF-1α directly suppresses FOXP3 induction, rather than indirectly inhibiting commitment through reprogramming toward alternative effector subtypes (10). In cancer, tumor hypoxia promotes an immunosuppressive TME by enriching FOXP3^+^ Tregs. Hypoxia stabilizes HIFs and induces CXCL12-CXCR4 signaling to recruit and retain Tregs within poorly perfused niches (50, 51). In lung cancer, hypoxia-driven CXCL12-CXCR4 signaling is well documented and supports preferential trafficking of Tregs into the TME (52, 53). Consequently, tumors display elevated Treg: CD8^+^ ratios, which correlate with poorer outcomes. Within these hypoxic niches, Tregs maintain suppressive function, a phenotype that may be supported by HIF-2α-dependent programs rather than by acute HIF-1α-driven effects on iTreg induction, thereby facilitating immune evasion and tumor progression (28).

## Treg responses in hypoxic pathological contexts

Despite growing insight into how hypoxia and HIF-1α shape Treg biology, clinical evidence remains limited and often heterogeneous. Nevertheless, current data begin to clarify how distinct hypoxic patterns influence immune regulation. The following sections synthesize available findings on Treg adaptation in chronic hypoxia, exemplified by COPD, in intermittent hypoxia characteristic of OSA, and in clinical settings such as COVID-19, where oxygen availability and immune regulation are profoundly altered.

### Tregs in chronic sustained hypoxia: evidence from COPD

COPD is a progressive respiratory disorder characterized by persistent airflow limitation, airway remodeling, and chronic inflammation. Sustained oxygen deprivation contributes to disease progression by promoting continuous activation of hypoxia-inducible pathways, oxidative stress, and inflammatory signaling (54). The role of Tregs in COPD remains incompletely defined, with studies reporting highly variable results across patient cohorts and anatomical compartments. Some reports describe an increased proportion of circulating CD4^+^CD25^+^CD127^-^FOXP3^+^ Tregs in COPD patients compared with healthy non-smokers, but this difference often disappears when compared to non COPD smokers, suggesting that part of the observed immune variation may be related to smoking rather than the disease itself (55, 56).

In contrast, other groups have recently demonstrated a decline in Treg frequency and FOXP3 expression, particularly in moderate to severe COPD (12). Hou et al. found no significant differences in total CD4^+^CD25^+^FOXP3^+^ Tregs among COPD patients, smokers, and controls but observed a phenotypic redistribution marked by reduced resting and activated Tregs (CD25^++^CD45RA^+^ and CD25^+++^CD45RA^-^) together with an increase in cytokine-secreting, non-suppressive subsets (Fr III: CD25^++^CD45RA^-^), indicating impaired regulatory function (57). Similarly, Sileikiene et al. reported lower Treg proportions in patients with advanced COPD (GOLD III-IV) compared with those with milder disease and non COPD smokers (58). During acute exacerbations, the pattern remains inconsistent: while some studies describe a transient reduction in circulating Tregs, Xiong et al. observed the opposite trend, with a temporary expansion during exacerbations (59).

A recent meta-analysis by Jalalvand et al., which integrated 24 studies comparing COPD patients and healthy controls, found no significant differences in the overall proportion of circulating Tregs. However, the authors noted substantial heterogeneity in study design, disease stage, and Treg phenotyping, which may have obscured subtle alterations in specific subsets. They proposed that, despite stable numbers, functional impairment and phenotypic instability of Tregs could contribute to the persistent inflammation observed in COPD, consistent with evidence of reduced suppressive subsets and increased proinflammatory Treg phenotypes reported in individual studies (60).

### Tregs in intermittent hypoxia: Evidence from obstructive sleep apnea

OSA is a prevalent disorder characterized by recurrent episodes of upper airway obstruction during sleep, leading to transient cessations (apneas) or reductions (hypopneas) in airflow (61). These events result in IH, sleep fragmentation, and sympathetic activation, which together promote oxidative stress, systemic inflammation, and metabolic dysregulation (62). Over time, this pattern of cyclic intermittent hypoxia contributes to cardiovascular, metabolic, and immune alterations, positioning OSA as a clinically relevant model of chronic intermittent hypoxia (63).

Evidence on the impact of chronic intermittent hypoxia on Tregs in OSA remains limited and largely derived from small clinical and experimental studies. In non-obese children with OSA, Ye et al. reported a significant reduction in circulating CD4^+^CD25^+^FOXP3^+^ Tregs accompanied by increased Th17 cells and a higher Th17/Treg ratio, which correlated with disease severity, inflammation, and HIF-1α expression; adenotonsillectomy reversed these changes, restoring Treg frequencies and lowering proinflammatory cytokines (64). Anderson et al. similarly demonstrated that overall tonsillar Tregs may be unchanged, but a decrease in FOXP3^+^ regulatory subsets within tonsillar tissue, particularly CD8^+^FOXP3^+^ Tregs, alongside elevated IL-17 and IL-1β expression, indicating local immune dysregulation and Th17-driven inflammation in pediatric OSA (65). In adults, Shen et al. found a similar imbalance, with decreased Tregs and elevated Th17 cells in untreated OSA, both of which normalized following continuous positive airway pressure (CPAP) therapy, together with reduced HIF-1α, IL-6, and IL-17 levels (47). Supporting these observations, a Mendelian randomization analysis by Ye et al. identified a protective association between higher levels of activated and secreting Tregs and reduced OSA risk, suggesting that regulatory T cell activity may represent a compensatory mechanism to counteract inflammation induced by IH (66).

### Tregs in COVID-19

In COVID-19, profound, often silent hypoxemia accompanies intense pulmonary inflammation and cytokine storm, creating a chronically sustained hypoxic environment (67). Among all immune cell populations perturbed by SARS-CoV-2, Tregs stand out as one of the most consistently disrupted, revealing a profound and stage-specific imprint of COVID-19 on immune regulation (68–70). Acute SARS−CoV−2 infection causes a rapid loss of circulating Tregs, particularly the naïve and central−memory subsets, and this decline closely parallels the severity of systemic inflammation (68, 71). Aquino et al. further demonstrated that this depletion is long-lasting: even 12 months after infection, total Treg numbers remain below those of healthy donors, with only partial recovery of CD39 and persistent suppression of CD73 expression (70). In parallel, Caldrer et al. showed that reduced Treg counts at hospital admission have strong prognostic value, with baseline levels predicting clinical worsening during hospitalization (71). Additional work by Salehi Khesht et al. indicates that hospitalized and ICU patients display the most pronounced reductions in Treg frequency, accompanied by diminished FOXP3 expression and impaired IL-10 production, both inversely correlated with inflammatory markers such as CRP and ferritin (72). In severe disease, expanded Tregs adopt a dysfunctional, tumor-like phenotype with IL-32 expression, decoupling numerical increases from suppressive efficacy (68, 69).

## Discussion

Across hypoxic settings, Tregs emerge as key intermediaries linking oxygen availability to the regulation of immune responses. The evidence reviewed here indicates that hypoxia influences Treg abundance, stability, and functional specialization through mechanisms that reflect both the intensity and the temporal pattern of oxygen deprivation. These observations support the concept that Tregs are highly sensitive to fluctuations in metabolic and microenvironmental cues, positioning them as active participants in the immune regulation triggered by hypoxia rather than passive targets of tissue stress. Important uncertainties, however, remain unresolved. Most studies rely on peripheral blood measurements, which only partially capture the behavior of tissue-resident Tregs operating within profoundly hypoxic niches. Variation in Treg phenotyping strategies and inconsistent definitions of functional subsets also limit the comparability of existing reports. Mechanistically, the dual actions of HIF-1α underscore the complexity of Treg responses: depending on the context, HIF-1α can stabilize FOXP3 expression and support suppressive function or, conversely, promote lineage instability and inflammatory skewing. This suggests that metabolic and epigenetic pathways intersect with oxygen sensing in a more nuanced manner than previously appreciated. Taken together, current findings highlight a need for a more integrated understanding of how hypoxia shapes Treg biology across diseases. Given the heterogeneity of available studies, **Table 1** summarizes key findings across hypoxic clinical settings, highlighting shared alterations in Treg phenotype as well as context-specific adaptations linked to distinct patterns of oxygen deprivation. Future work will require standardized characterization of Treg subsets, incorporation of tissue-level analyses, and the application of high-resolution metabolic and epigenomic profiling. Such approaches will be essential to determine whether modulating Treg responses to hypoxia can be leveraged as a therapeutic strategy in conditions characterized by chronic sustained or chronic intermittent oxygen deprivation.

**Table 1:** Summary of key studies evaluating Treg alterations across hypoxic conditions.

Researchers	Context or condition	Population or experimental model	Key Treg findings
Tregs and HIF-1α			
Shi 2011; Dang 2011 (35, 45)	HIF-1α regulation (central mechanism)	Mouse and *in vitro*	HIF-1α promotes glycolysis, ↑ Th17, and drives FOXP3 degradation → Treg instability
Clambey 2012 (48)	Physiologic mucosal hypoxia	Inflamed mucosal tissue (mouse)	Low O_2_ physiologic environments may ↑ FOXP3 and enhance suppressive function
Lee 2015 (46)	VHL-HIF-1α Treg alteration	Genetically modified Tregs (mouse)	Sustained HIF-1α activation → ↓ FOXP3, ↑ IFN-γ; silencing restores function
Chronic obstructive pulmonary disease			
Kalathil 2014; Vargas-Rojas 2011; Hou 2020; Sileikiene 2019 (55–58)	COPD	COPD patients, smokers, non-smokers	Total Tregs normal or ↑ but dysfunctional: ↓ suppressive Tregs, ↑ non-functional Tregs, ↑ Th17
Hou 2020; Sileikiene 2019 (57, 58)	Treg subsets in COPD	Peripheral blood and bronchial mucosa	↓ resting/activated Tregs; ↑ pro-inflammatory Treg-like populations (Fr III)
Xiong 2022 (59)	COPD exacerbation	Patients in exacerbation vs stable state	Variable: transient ↓ or ↑ in Tregs depending on clinical phase
Obstructive sleep apnea			
Ye 2015 (64)	Pediatric OSA (blood)	Children with obstructive sleep apnea	↓ Tregs, ↑ Th17; ↑ Th17/Treg ratio correlates with severity
Anderson 2014 (65)	Pediatric OSA (tonsils)	Tonsillar tissue	↔ Tonsillar Tregs (CD4^+^CD25^+^FOXP3^+^)
Shen 2024 (47)	Adult OSA (before/after CPAP)	Adults with OSA	↓ Tregs, ↑ Th17, ↑ HIF-1α; CPAP reverses alterations
Ye 2012 (66)	Adult OSA (blood)	Adults with OSA vs healthy controls	↑ Th17 cells, ↑ IL-17/IL-6, ↑ RORγt; ↓ Tregs (CD4^+^CD25^+^FOXP3^+^), ↓ FOXP3 mRNA
Tregs in COVID-19			
Galvan-Peña 2021 (69)	Acute COVID-19	Mild, moderate, severe patients	↓ naïve/memory Tregs proportional to severity; FOXP3^high dysfunctional Tregs with IL-32
Caldrer 2021 (71)	Hospitalized COVID-19	Hospitalized patients	Treg levels predict clinical worsening
Salehi Khesht 2021 (72)	Severe COVID	ICU patients	↓ FOXP3, ↓ IL-10; strong Treg loss
Aquino 2024 (70)	Post-COVID persistence (12 months)	Recovered patients	Incomplete recovery: CD39 ↑ partial; CD73 remains ↓

The symbols indicate: ↓ decreased/downregulated; ↑ increased/upregulated; → leads to/results in; ↔ bidirectional relationship/mutual association (context-dependent).
